# POLICY SERIES: ARE WE THERE YET? MEASURING PROGRESS IN ACHIEVING POLICY GOALS FOR FAMILY CAREGIVERS

**DOI:** 10.1093/geroni/igad104.1641

**Published:** 2023-12-21

**Authors:** Pamela Nadash, Rani Snyder

## Abstract

The RAISE Family Caregiving Advisory Council, created under the Recognize, Assist, Include, Support, and Engage (RAISE) Family Caregivers Act (2018) has been tasked to support the Secretary of Health and Human Services in developing a national family caregiving strategy, published in 2022 as its National Strategy to Support Family Caregivers. One key concern identified by advocates and others is the accountability question: how can we hold policymakers at all levels of government accountable for achieving the policy goals articulated in the National Strategy? This symposium discusses that issue by first providing an overview of a key mechanism for state accountability, AARP’s State Scorecard on Long-Term Services and Supports, which identifies support for family caregivers as one of its four dimensions. Susan Reinhard, PhD, Senior Vice President at AARP and Director of its Public Policy Institute, will describe the history, goals, and future of the Scorecard’s family caregiver component. Salom Teshale, PhD, from the National Academy for State Health Policy will describe how states are focusing their energies in current actions supporting family caregivers, while Mike Wittke, MPA, of the National Alliance for Caregiving will discuss how federal actions might be tracked and assessed. Drawing on research supporting the RAISE Advisory Council, Eileen J. Tell, MPH will suggest additional dimensions that could be integrated into the assessment of progress. Rani Snyder, MPA, of the John A. Hartford Foundation will act as discussant.

